# Hospital and Institutional News

**Published:** 1911-10-14

**Authors:** 


### HOSPITAL AND INSTITUTIONAL NEWS.

### AN EMPLOYER'S BEQUEST TO WEST BROMWICH HOSPITAL.

A VERY interesting bequest has been made by Mr. John Homer Chance, which virtually will leave the West Bromwich District Hospital residuary legatee of several thousand pounds. Through the medium of Mr. G. F. Chance, Mr. F. G. Hancock, the secretary of the hospital, has been informed that by deed dated February 5, 1898, Mr. John Homer Chance gave £4,000 to Messrs. Chance Bros. and Co., Ltd., in trust to apply the income in paying pensions to workpeople of the firm, the surplus income to be carried forward or given to the District Hospital, West Bromwich. This condition was to continue for ten years, when the firm might extend the time for paying pensions, but without granting any new pensions. When all pensions come to an end the fund will, if certain conditions are fulfilled, be handed to the hospital. As investments had to be made when all securities were at the highest point, the annual income at present amounts to about £133. He enclosed a cheque for £50 as the first payment on account of surplus income. This is interesting as an example of the way in which many of the large employers of labour recognise that the least controversial way of doing their duty to those whom they employ is to support the local hospitals. Both working-men and employers benefit by the added number of working days a year that the skilled treatment in hospitals has procured for them. Under the proposed Insurance Bill the memories of both might deteriorate.

### AN OFFICIAL WITH SEVEN ALIASES.

A WANT of principle seems to apply to institutional secretaries, for in looking through a list we find the duties of that official disguised under no less than seven different aliases. At some hospitals he is known as "the clerk"; at others, "the superintendent." The titles of general manager, curator, registrar, and house governor equally seem to suffice. It would be idle to pretend that these names really represent subtle distinctions of duties, although, as in all growingly responsible posts, an institutional secretary's duties have multiplied enormously of recent years. What some of these names really represent is a change not of duties, but of status. Originally a hospital secretary was, like his institution, a thing to be passed by. Then his responsibilities grew before his remuneration. A better class of man was wanted and found, but the old name and salary still persisted. This was galling enough, and produced a worthy struggle by the best officials for a due recognition of the increased value of their work. These pioneers were successful and raised the status and advantages of their profession all round. One or two of the above aliases are all that remain to remind us of a different state of things, though the old names themselves now share the honours that are often represented at other institutions by more imposing titles.

### THE HOSPITAL SATURDAY FUND.

The quarterly meeting of the Board of Delegates of the Metropolitan Hospital Saturday Fund, was held on Saturday evening, at the Delegate Hall, Hearts of Oak Buildings, Euston Road. Mr. W. F. Chalkley presided in the unavoidable absence of Sir Savile B. Crossley, Bt., (chairman of the Fund). A vote of condolence with the widow and family of the late Mr. W. G. Bunn, a former Secretary, was unanimously adopted on the motion of the Chairman, seconded by Mr. Thomas Bevan. The following letter (addressed to the Secretary), was read from Sir Savile Crossley: "I regret to be unable to attend, being engaged in Hampshire, especially as I should like to be present at the moving of the resolution of condolence with the family of your distinguished predecessor in office—Mr. Bunn. I note from the *Daily Mail* of September 30, that Sir H. Burdett is reported to have stated at the British Hospitals Association meeting in Manchester on September 29, that the Hospital Saturday Fund was not in a satisfactory state. It is therefore the more necessary to point out now that the Hospital Saturday Fund, in spite of the introduction of the Insurance Bill, is in a more satisfactory state than ever before, the income being greater than ever before, and the working expenses steadily declining as you all know. I trust that the *Daily Mail* will be kind enough, therefore, to give the same publicity to this correction, as it has already given to the statement which has probably been incorrectly reported from the speech of my friend Sir Henry Burdett. I enclose copy of the paper named." The Secretary (Mr. Davis), said he had written to Sir Henry Burdett, and asked him whether he had been correctly reported, and whether that statement referred to the Hospital Saturday Fund (London). Sir Henry had replied as follows: "I am wrongly reported in the *Daily Mail*, as you have probably seen from the concluding words of the paragraph. I was speaking of the unsatisfactory state almost everywhere, of 'Hospital Sunday,' as a movement, and with the desire of re-creating and renewing these organisations throughout the country. The reporter fell into the mistake because 'Hospital Saturday' and 'Hospital Sunday' in Manchester are, I understand, worked by the special Hospital Saturday Fund Committee." Sir Henry Burdett, in concluding a note which he had written to the *Daily Mail*, said "the matter is important, because everywhere 'Hospital Saturday' is improving whilst 'Hospital Sunday' is languishing or even manifesting symptoms of decay."

### DEATH OF A NOTED SHANGHAI SURGEON.

THE death occurred last week of Dr. Louis Stromeyer Little at Bletchingley, Surrey, in his seventy-first year. Dr. Little had a career that was well filled with interest and activity. Not long after qualifying he engaged in the campaign in Schleswig in 1864, and did a great deal of surgical work among the wounded after the storming of Duppel. In 1866 he became a Fellow of the Royal College of Surgeons, and during the following ten years he held surgical appointments at the Orthopædic Hospital and the St. Mary's Dispensary, and soon became Surgeon to the London Hospital. This, however, he gave up about 1876, when he proceeded to China, where he remained for twenty years in Shanghai. He soon acquired a great reputation and a large practice, and was appointed Surgeon to the Shanghai General Hospital. Dr. Little returned to England about 1896 and lived in retirement at Bletchingly, where his death has occurred after a long illness. Among his diplomas must be mentioned that of the M.D. of the University of Kiel, and it is interesting to note that he was a member of the Astronomical Society.

### PERSONAL SERVICE.

By the retirement of Mr. B. Rix from the surgical staff of the Tunbridge Wells General Hospital, the institution has to deplore the loss of one of its most able workers. In 1872 Mr. Rix was appointed house surgeon, and served in that capacity for over two years, and on the retirement of his brother from the active staff in 1900 he was elected a surgeon; since when Mr. Rix has been most untiring in his work for the patients under his care and for the hospital generally. The committee accepted his resignation, which was due to the age limit being reached, with much regret, and to mark its deep appreciation of Mr. Rix's services unanimously elected him a consulting surgeon of the Hospital.

### THE FIRST MEDICAL OFFICER OF HEALTH.

In the person of Dr. Theodore Günther there has just past away the first medical officer of health appointed under the Public Health Act. For forty years Dr. Günther held the joint appointments of M.O.H. for Hampton Wick and Teddington, to the former of which he was appointed in 1865. When he retired he was the senior district and Poor Law medical officer in the United Kingdom. Dr. Günther was born in Stuttgart and studied at the University of Tübingen, where he took the M.D. degree in 1857. After serving as lieutenant in the Würtemburg Army, he was called to England, and through the favour of the Prince Consort was appointed physician to Prince Leopold, with whom he spent a long time in Cannes. In 1865 he obtained an English qualification, after having settled in Hampton Wick, and there continued to practise for over forty years.

### A NOTED ZOOLOGIST OF THE I.M.S.

Lieut.-Colonel Jayakar, whose death recently has been reported from Bombay, was one of the first Indians to pass into the Indian Medical Service. In 1867 he became the first holder of the travelling scholarship founded at the Bombay University to encourage Hindu graduates to complete their training in this country. Having studied at the Grant Medical College, he came to London and obtained the M.R.C.S. and L.R.C.P. diplomas in 1867, and soon after entered the Indian Medical Service. As residency surgeon at Muscat for nearly thirty years, he employed his leisure in getting together a large zoological collection. He sent many specimens to this country, notably a most valuable collection of mammals. An important discovery of his was that of a new goat, which has been named after him; he also discovered a new hare, two new species of lizards, and two new snakes. A large zoological lexicon was completed by Colonel Jayakar, which is, however, not yet completely through the press.

### PRACTICAL GRATITUDE.

There are still living, though probably the number is steadily diminishing, medical men to whom it has happened that grateful patients have complained of the smallness of their bills, and have insisted on paying larger sums than those requested. In modern days this exuberance of gratitude seems to have disappeared before the commercial spirit of the age; but it is edifying to know that patients do still occasionally express gratitude for medical services in deeds as well as words. From a trustworthy source we learn that a gentleman of means, whose wife has just passed successfully through a very long and dangerous illness, intends to mark his appreciation of the devotion shown by the general practitioner in charge of the case by founding three scholarships at Cambridge for young medical men. According to the terms of the foundation, these scholarships will bear the name not of the founder but of the said practitioner, who will thus achieve an immortality which seldom falls to the lot of the rank and file of the medical profession. While this handsome gift to the University and to medical education reflects great honour and credit on the donor, we desire to congratulate him even more upon the happy thought which has inspired him in the selection of a name for the scholarships. The whole spirit of his gift is one which is worthy of emulation by wealthy members of the public, and we earnestly trust that this good example may find numerous imitators.

### A CURIOUS ADVERTISEMENT.

The following advertisement appeared last week in a prominent London daily: "Medical man, about to retire, wants an appointment which will occupy him during some hours daily." We are afraid that this hardly implies that the advertiser has been, or has immediate prospects of being, placed upon any institution's honorary consulting staff. It would rather strike the public that a medical man has found his profession unable to afford him a living wage. If this interpretation be correct it is highly probable that there are many medical men in the same straits, so small, taking the number of those qualified, is the average income of the profession.

### PROPOSED LABORATORY FOR EUGENICS.

The personal work and careful posthumous directions of the late Sir Francis Galton will probably do more to attract public support to his desired laboratory than the object itself. The appeal for £15,000 to build the laboratory, which has now been issued by the University of London, justly points out that Sir Francis Galton's wish that the capital left by him for endowment purposes should not be frittered away on building and equipment has been fulfilled, and we are very glad of it. No doubt because of the difficulties attending eugenic experiments, a laboratory building will be especially useful. The public, however, undeniably has let certain self-appointed exponents of this science prejudice it against eugenics, and it will be the power of the late Sir Francis Galton's reputation rather than the proposed laboratory which will gain the support of the public in this matter. Plans for the laboratory have been prepared by Professor F. M. Simpson, F.R.I.B.A., and can be seen at University College, Gower Street.

### WHAT TO DO WITH SMALLPOX HOSPITALS.

All over the country the local authorities are being worried by the county authorities either to provide isolation hospitals or to adapt existing hospitals for smallpox to other purposes. The latest example that comes to notice is the reported necessity for an isolation hospital for the use of the Ripon Rural District, and Dr. Collier, M.O.H., has reminded the District Council that the Lark Hill Hospital has never been used for anything but smallpox, and that "there were three cases in 1902 and none since." The question of the adaptability of the disused building is now under consideration. There is little doubt that if the proposed new sanatoria are to be built an attempt will be made to save money by "adapting" similar empty smallpox hospitals for consumptives. It seems generally believed that at present there are far more smallpox hospitals existing than the number of smallpox cases warrant. While congratulating ourselves on the fact, care must be taken to avoid the false economy of attempting to adapt obsolete buildings to new and different purposes. This warning is all the more necessary as district smallpox hospitals have not always kept abreast of the modern standard of building and of equipment.

### DR. HUGHLINGS JACKSON.

Dr. John Hughlings Jackson, F.R.S., Consulting Physician to the London Hospital and to the National Hospital for the Paralysed and Epileptic, died last Saturday in London. It is a long cast back to the days, about 1855, when Dr. Jackson began his studies as apprentice to a surgeon in York. After qualifying in London, he returned to York as House Surgeon to the dispensary, till, through the advice of his friend Sir Jonathan Hutchinson, whom he had met at "Bart.s," he was persuaded in 1859 to return to the capital. Dr. Jackson will be remembered for leading the way to the discovery of the position of the speech centre in the left frontal convolution of the brain, and for associating with definite lesions what is now known as 'Jacksonian " epilepsy. He had a large consulting practice in London, and was a frequent contributor to medical journals and to the Transactions of various societies. Always somewhat a recluse, the early death of his wife deepened his retiring tendencies, and he was not widely known outside his intimate circle. Living in a big house by himself confirmed his dislike of society and perhaps conduced to the deafness and other signs of old age which he had lately shown.

### ANOTHER M.P. AND HIS SALARY.

To the Mayor of Hastings has been sent a letter from Mr. Arthur du Cros, on the subject of the remittances the latter has received in respect of his salary as member of parliament. As Mr. du Cros is anxious that his present relations should continue without personal benefit to himself directly or indirectly he has asked the Mayor to nominate, and if possible to preside over, a small committee to administer the money as a special fund for the general welfare of the borough. We trust that the secretaries of the hospitals in Hastings will be fully alive to this splendid opportunity of pressing their claims to a share in this new and unobjectionable form of State aid for their institutions. It is very gratifying to find that the excellent example set by Mr. Arthur Fell, at Great Yarmouth, is being followed in other parts of the country. One of the latest contributions of this kind is that of £100 from Mr. G. A. Touche, M.P. for North Islington, who has forwarded this sum to the Great Northern Hospital, Holloway Road, with an intimation that it may be regarded as an annual donation as long as present conditions continue.

### THE NELSON MEMORIAL HOSPITAL AT MERTON

The building of the Nelson Hospital for the Wimbledon and Merton district is now proceeding and serious efforts are being made to collect the remaining three to four thousand pounds, which are required to open the institution free from debt. Colonel Mitchell, C.B., chairman of the organising committee is appealing for funds for this purpose. The building is being erected at Merton as a memorial of Nelson's residence there from 1801 to 1805, and is to take the place of the existing South Wimbledon, Merton, and District Cottage Hospital, founded in 1900. The committee have £7,000 in hand, and a grant of £1,000 has been made from King Edward's Hospital Fund. Accommodation will be provided for forty patients. We have every confidence that the inhabitants of the flourishing borough of Wimbledon will see to it that when this memorial to one of the greatest of our national heroes opens its doors for its beneficent and much needed work, it will be free from the crippling incubus of a debt.

### COMMONSENSE AND CHARITY.

We note with satisfaction the public announcement that the St. John's House of Rest at Mentone will reopen on November 1, and that a very practical benefaction has been made to it which will much enhance its sphere of usefulness. This convalescent institution, which has done useful work for more than thirty years, was founded for the clergy and the professional laymen, who for a small sum are received after illness or breakdown and cared for in the House until they are once more fit to return to their occupations. The particular contribution to which we have just referred is from an anonymous donor, who has provided the sum of £100 to be expended in assisting professional laymen in meeting the expense of reaching Mentone. A more helpful way of ear-marking such a donation would be hard to devise, and the anonymous benefactor is to be congratulated both upon his liberality and his common-sense. Naturally for those whom the House is intended to benefit, the expense of the journey is a material consideration, especially as an invalid can often travel only if the journey is rendered as comfortable as possible by such adventitious luxuries as first-class railway travel, or even in some cases by a sleeping-berth. At first sight it seems a little invidious to restrict the money to laymen; but probably the reason for this is that the Committee of St. John's House have already at their disposal means of procuring similar financial help for clergymen who require it.

### OUT-PATIENT EXTENSION AT PADDINGTON GREEN.

When the children's hospital on Paddington Green closed its doors on June 3 in order to carry through the extension of its out-patient department we published some description of the new scheme. It may now be well to add that the opening day has been fixed for Thursday November 16, and that H.R.H. Princess Louise, Duchess of Argyll, has graciously consented to perform the ceremony. Two things are interesting about this new development, the first is the way in which the architect Mr. Alan E. Munby has tackled the problem of conversion and addition, for the old out-patients' hall has been retained for the dispensary, and the second is that the whole department will be brought up to date by the appointment of an official almoner, who will carry on and extend the work that has gone on so steadily and unobtrusively in the hands of Miss Stubbs.

### KING VICTOR EMMANUEL AND ITALIAN INCURABLES.

The first bed of a hospital for incurables in Casale has been dedicated to King Victor Emmanuel II. Casale which is on the Po, was formerly the capital of the Monferrato duchy, and up to last month was, we believe, without any institute for the relief of chronic invalids. The city's mayor, however, enforced the necessity of founding a suitable hospital for incurables. He not only appealed to the rich but obtained a subsidy from the commune. Finally a large but inadequate sum was raised for the purpose. On the occasion of the visit of the Sovereign during the past manœuvres, Victor Emmanuel gave 5,000 lire (£200) for the benefit of the city, which the mayor added to what he had been able to collect, and with local support, the site being given, he founded last month (September 21) the hospital of the incurables, dedicating the first bed to the name of Victor Emmanuel III. in recognition of the assistance that the King had given. We do not know whether Casale is to be congratulated more upon the enterprise of its mayor, Signor Tavallini, or upon the foundation of its new institution.

### FRENCH SURGEON'S CLAIM TO SUPERSEDE PHYSICIANS.

At the inaugural sitting of the French Association of Surgery last week at the Faculty of Medicine, M. Segond, in the course of his speech, referred with unconcealed emotion to the two deceased French surgeons, Dr. Guinard, who was assassinated in the court of the Hôtel Dieu, and Charles Nélaton, of famous family, who was M. Paul Segond's intimate friend. The speaker went on to say that operators are always reproaching medical practitioners for confiding their patients too late to them when there have already appeared the first signs of such diseases as peritonitis. The remedy offered by M. Segond was that the operators should be able to be physicians and surgeons at the same time. Whatever physicians may be inclined to think of this exaggerated claim, it has been pointed out very often by surgeons that the most typical and increasingly frequent function of the modern physician is the administration of anæsthetics. Whatever the advances of modern surgery, there is little doubt that medicine has suffered from the ease with which anæsthetics enable surgeons to operate at the present time.

### HOSPITALS AND TELEPHONE CHARGES.

The issue of a report by the Council of the British Hospitals Association on its efforts to secure reduced charges for hospitals using telephones, will be awaited with considerable interest by hospital workers. Similar direct State or local aid in the matter of grants for medical education, for the hospital treatment of school children, and in the payments made by the Poor Law for those serious cases which it sends from time to time for treatment in the voluntary hospitals can be found readily enough. The reduction of rating assessments by local authorities is perhaps the commonest form of such indirect municipal aid. Evidently there are plenty of precedents, but probably one of the difficulties in the matter is that the Government paid such a high price for the recent purchase of the telephones that it will be more inclined to raise than to lower its charges. We hope not, and if the Council's efforts to secure a reduction are unavailing, at least it will be curious to see on what grounds their propositions are rejected. The attempt is a solid example of the good work done by this Association, which worries its members for meetings and for subscriptions extraordinarily seldom, while it tackles such difficult hospital questions as the above with commendable energy.

### THE KING EDWARD COT AT TUNBRIDGE.

The Tunbridge Wells General Hospital was visited on Friday, October 6, by the Mayor and Mayoress (Colonel and Mrs Sladen), when the tablet of the King Edward Memorial Cot in the Queen

Victoria Memorial Ward was unveiled. A large number of prominent residents and supporters of the Hospital assembled in the board-room to welcome his Worship and the Mayoress, and accompanying them, made a complete tour of the building. At four o'clock the company made their way to the Queen Victoria Memorial Children's Ward for the unveiling ceremony. After a brief introductional speech by Mr. F. Wadham Elers, the Chairman of the Hospital, the Mayoress unveiled the tablet, which was engraved with the arms of the borough and the following inscription:—"This Cot is endowed by a fund inaugurated by the Mayor of Tunbridge Wells in memory of his Gracious Majesty King Edward VII." The company afterwards partook of tea in the Out-Patient Waiting Hall.

### THE FIRST MEDICAL MAN TO BE LORD MAYOR OF LONDON.

For the first time in its history the City of London's Chief Magistrate is a member of the medical profession. Sir Thomas Boor Crosby, who has been elected to the office of Lord Mayor for the ensuing year, has already reached the mature age of eighty-one. He is one of the oldest practising medical men in the kingdom, having qualified in 1852 at a very early age. He has been a Fellow of the Royal College of Surgeons since 1860, and obtained the M.D. of St. Andrews in 1862. To St. Thomas's Hospital belongs the honour of being his *alma mater*, and no doubt during the year this hospital will be the scene of some ceremonial incidents celebrating the occupation by a St. Thomas's man of the highest office the City of London has to offer. Sir Thomas Crosby has been recipient of many foreign orders, including that of the Légion d'Honneur of France and the Order of the Rising Sun of Japan.

### NATIONAL INSURANCE BILL.

The Conference held at the Treasury on October 9, at which representatives of the British Medical Association and the friendly societies discussed with the Chancellor of the Exchequer certain aspects of the National Insurance Bill, was of a strictly private character. It is understood, however, that the time was mainly occupied in considering the objections of friendly societies to some of the amendments which were made in Clause 14 during the committee stage and that the conference will be resumed on October 16. If, as is thought possible, the friendly societies and the British Medical Association arrange a settlement of their difficulties, the prospects of the Bill will be much brighter. It is interesting to note that the chairman of the Labour Party has stated that the party will adopt the Bill. A Parliamentary paper has been issued which contains the official reports of deputations to the Chancellor of the Exchequer concerning the Bill. One of the reports deals with the deputation representing the British Hospitals Association, the Central Hospital Council of London, and the Hospital Saturday Fund for London which waited upon the Chancellor on July 25.

### CHANGES AT THE WEST END HOSPITAL FOR NERVOUS DISEASES.

Dr. David Findlay has just taken up his appointment as Medical Officer in charge of the Electrical Department at the West End Hospital. Dr. Findlay is M.D., C.M. of Edinburgh, and gained his doctorate by a thesis on the application of electricity to therapeutics. Mr. F. F. Middleweek, L.R.C.P., L.R.C.S., L.F.P.S.G., has been appointed director of Mechano-Therapeutics for Male Patients. Mr. Middleweek was a civil surgeon in the Boer War and has studied in Stockholm. Miss C. L. Bedingfield has been appointed Massage Sister and Teacher. Miss Bedingfield was trained at St. George's Hospital, and holds the C.M.B. certificate and the certificate of the Incorporated Society of Trained Masseuses. She has recently been acting as assistant to the Vice-chairman and Examiner of that society in massage classes. The hospital trains for its own certificate in electrical and massage treatment and also for that of the Incorporated Society.

### SAMARITAN FREE HOSPITAL.

The Samaritan Free Hospital for Women, Marylebone Road, is to have a new home for the nurses, and the building of the proposed premises is now in hand. This step was found necessary owing to the old Home, now demolished, adjoining the Hospital, having become dilapidated and too decayed to be repaired. It will involve an outlay of over £3,000.

### A HOSPITAL ARCHITECT AS MAYOR.

Mr. Frank Wills, the newly chosen Lord Mayor for Bristol, is an architect who has done a considerable amount of hospital construction, renovation, and extension work in his native city, which is now proud of the selection for mayoralty from a family whose beneficence has conferred so many obligations on her. A good many references, calculated slightly to mislead outsiders, have been made to him as the brother of the late Mr. H. O. Wills, whose generous bequest to the Bristol General Hospital (as well as bequests to other institutions of more or less similar nature), has been indicated in these columns. Mr. Frank Wills is the half-brother of the deceased gentleman, and, in point of fact, one of his younger brothers, Mr. Grahame H. Wills, is some years the junior of the late Mr. H. O. Will's eldest surviving son Mr. George Alfred Wills, who for many years past has been a specially generous supporter of the Bristol General Hospital, an institution to whose organisation he has devoted a considerable portion of valuable time in addition to liberal monetary contributions.

### THE SLEEPLESSNESS OF IN-PATIENTS.

It was hardly to be expected that any great result should arise from Sir John Bingham's recent attempt to relieve the patients in the wards of the Royal Hospital, Sheffield, from the street noises that make sleep practically impossible for them. He was promptly told that it was the hospital rather than the noise which should be shifted. There is no need to dispute this view, but in the meantime certain other facts should be remembered. Tramways apart, the chief cause of the noise in our streets is the fact that two forms of traction now traverse them. When all equipages have become autopages there will be less noise as well as less risk; and there are many people in Sheffield who would thank Sir John Bingham for attempting to have its cobbles replaced by macadam, wood, or asphalt. Northern cities are as much behind London and the Southern county towns in this respect as they are in front of them in their possession of repertory theatres, and other things. If a campaign on behalf of hospital patients could convert the cobbles of Sheffield and other industrial cities into wood or asphalt paving the noise nuisance would be diminished by one-half. The time of shifting the hospitals to the suburbs could thus be usefully filled.

### INSTITUTIONS AND CHILD-STUDY.

The programme arranged by the Child-Study Society, which meets fortnightly at 90 Buckingham Palace Road, includes several features which are of institutional interest and many which are of general sociological importance. Primarily these discussions, which are usually informative owing to the fact that many well-instructed members of the society participate in them, are intended to appeal to those who are interested in the problems of child-study, but even the ordinary institutional worker, especially if he be connected with a children's hospital or has himself any concern with the younger generation, will find these debates stimulating. On October 12 Mr. Montagu dealt with the George Junior Republic—a fascinating study in "pediatric politics," as the Americans say; on October 19 Dr. Kingsford will tackle the very debatable subject of "Co-education during Adolescence," and at the succeeding sessions questions such as the "Psychology of Speech," "Reading," and "Grammar," will be discussed. The annual subscription to the society is half a guinea, and the secretary is Mr. W. D. Mulford.

### AUTOPSIES ON HOSPITAL PATIENTS.

At the Trade Union Conference at Newcastle a resolution was put demanding legislation that no post-mortem examination should be made on hospital patients until forty-eight hours after death, and that the consent of the next-of-kin must be a condition. The latter proviso is, of course, always observed, it may be noted. Not the least of the qualifications of many hospital porters, in the eyes of the medical staff, has been their success in obtaining such consent by putting the matter sympathetically before the friends of the late patient. Indeed, we fear that the author of the above resolution must have been getting his ideas of medical men from the grotesque "scientists" who figure in the cheaper magazines; and this may be said without disrespect to working-class leaders, often men whose considerable native ability has unaided won them their position. Our hospital surgeons have no wish to emulate the famous Ruysch, and should have every assistance in their endeavours to investigate the seat of disease as nearly as possible. Hindrance to such endeavour is mere obscurantism, a fault particularly to be avoided by leaders of a movement which owes so much to the passing of the Education Act. It is as much obscurantism as any clerico-aristocratically inspired Russian ukase; a good example, indeed, of the tyranny into which, as we know, the morbid hatred of any kind of authority is apt to lead.

### MEDICAL MEN OF LETTERS.

A writer in the *Athenæum*, taking about three hundred authors given in a Dictionary of English Literature and examining them in respect of education and training, finds that seventeen qualified for or entered the practice of medicine. The category "author" is a rather wide one, as the names of medical men of letters given include the scapegrace poet Thomas Lodge, and the staid philosopher and contemporary historian Sir James Mackintosh, besides the great Locke and that glorious child of Apollo, Keats. It is curious to reflect how little medicine, as such, although it must have indirectly contributed to their experience of life, shows in the life-work of these men. An exception might be allowed in the case of Crabbe, whose Dutch painting of village life may well owe a little to his surgical apprenticeship in Suffolk. But eminence in medicine practically never goes along with poetic genius. One can hardly find instances beyond Arbuthnot (whose carelessness of literary fame has impoverished his reputation), Oliver Wendell Holmes, and, in our own day, the quondam medical student and ecstatic poet Francis Thomson, an enthusiastic appreciation of whom appeared in The Hospital of Christmas Day, 1909. To succeed conspicuously in the medical profession a man must have more taste for what our foremost living singer calls "dusty deeds" than is usually vouchsafed to poets.

### THIS WEEK'S DRUG MARKET.

The most important price alteration of the week is an advance in the value of cocaine; the rise, which was not unexpected, is mainly due to the increased cost of crude cocaine. Balsam Tolu is again dearer. Quicksilver has been slightly reduced in price. Hydrastinine is dearer. There is practically no alteration in the position of opium for which extremely high prices are still asked; it is not thought probable that the Italo-Turkish war will cause any material increase in the price of the drug which, if necessary, can be transmitted by the overland route. At the recent auction of drugs in Mincing Lane, Cape aloes sold at slightly cheaper rates but cardamoms and senna realised higher prices. The improvement in business in the drug market, which was recently noticed, is maintained and the general tendency continues to be in the direction of increased prices.

